# Proposed Maryland Dental Law

**Published:** 1896-04

**Authors:** 


### Editorial, Etc.

### PROPOSED MARYLAND DENTAL LAW.

A BILL entitled "An Act to repeal Article thirty-two of the Code of General Laws, entitled 'Dentistry, and to re-enact said article, with amendments."

SECTION 1. Be it enacted by the General Assembly of Maryland, that Article thirty-two of the Code of Public General Laws, entitled "Dentistry," be, and the same is hereby, repealed and re-enacted, so as to read as follows:

SEC. 2. It shall be unlawful for any person to practice dentistry in this State unless such person shall have obtained a certificate, as hereinafter provided.

SEC. 3. There shall be a State Board of Dental Examiners, which shall consist of six practicing dentists of recognized ability and honor, who have held regular diplomas for five years, whose duty it shall be to carry out the purposes and enforce the provisions of this Act. The members of said board shall be appointed by the Governor out of a list of nine dentists proposed by the Maryland State Dental Association, and chosen by a majority vote of the members of said association present at a meeting called for that purpose, of which meeting two weeks' notice shall be given. The term for which the members of said board shall hold their office shall be for six years, except that the members of said board first to be appointed under this Act shall be designated by the Governor to serve: One-third for a term of two years, one-third for a term of four years, and one-third for a term of six years, unless sooner removed by the Governor, and until their successors shall be duly appointed. In case of a vacancy occurring in said board, such vacancy shall be filled by the Governor from the list above mentioned. Any member of said board who shall be absent from two successive regular board meetings shall cease to be a member of it.

SEC. 4. Said board shall choose one of its members president and one secretary thereof, and shall hold regular meetings in May and November of every year, and special meetings, as occasion may require. A majority of said board shall at all times constitute a quorum, but a less number may adjourn from time to time; the proceeding thereof shall at all reasonable times be open to public inspection. The board shall make a report of its proceedings to the Governor by the first day of December in each year.

SEC. 5. Any person twenty-one years of age, who has graduated at, and holds a diploma from, a university or college authorized to grant diplomas in dental surgery by the laws of any one of the United States, and who is desirous of practicing dentistry in this State, may be examined by said board with reference to qualifications, and, upon passing an examination satisfactory to said board, his or her name, residence or place of business, shall be registered in a book kept for that purpose, and a certificate shall be issued to each person. Any graduate of a regular college of dentistry may, at the discretion of the examining board, be registered without being subjected to an examination.

SEC. 6. All certificates issued by said board shall be signed by its officers and bear its seal.

SEC. 7. A temporary certificate for a specified time may be issued by the officers of said board to any applicant holding a regular dental diploma duly registered by a board of dental examiners created by the laws of any one of the United States, but no such certificate shall be issued for any longer time than until the next regular meeting of the board. The fee for this temporary certificate shall be five dollars.

SEC. 8. Transcripts from the aforesaid book of registration, certified by the officer who has the same in keeping, with the seal of said board of examiners, shall be evidence in any court of this State.

SEC. 9. A fee of ten dollars shall be paid to the secretary of the board by any applicant for registration, which money shall be used towards paying the expenses of the board.

SEC. 10. Every person shall be said to be practicing dentistry within the meaning of this Act, who shall, for a fee, salary, or other compensation, paid either to himself or to some one else for services rendered, perform operations or parts of operations of any kind pertaining to the mouth, treat diseases or lesions of the human teeth or jaws, or correct mal-positions thereof.

SEC. 11. It shall be unlawful for any person not holding a regular diploma to assume the academic title of "doctor."

SEC. 12. Any person who shall violate any of the provisions of this Article shall be deemed guilty of a misdemeanor, and, upon conviction thereof, in any court having criminal jurisdiction, shall be fined not less than fifty dollars nor more than three hundred dollars, or be confined not more than six months in the county jail, or if the conviction takes place in Baltimore city in the Baltimore city jail, in the discretion of the court. All fines received under this Act shall be paid into the common school fund of the city or county in which such conviction takes place.

SEC. 13. Nothing in this Article shall be so construed as to interfere with the rights and privileges of resident physicians and surgeons, and dental students operating under the immediate supervisions of their instructors in dental infirmaries of dental schools chartered by the General Assembly of Maryland.

SEC. 14. And be it enacted, that nothing in this Act shall prevent, or be so construed, as in any way to hinder the prosecution or punishment of any person who may have offended against any of the provisions of said Article thirty-two of the Code of Public General Laws, or against any of the provisions of any of the Acts of Assembly of which the same was a codification.

SEC. 15. And be it enacted, That this Act shall take effect from the date of its passage.

